# Behavioral consistency of competitive behaviors and feeding patterns in lactating dairy cows across stocking densities at the feed bunk

**DOI:** 10.3389/fvets.2024.1302573

**Published:** 2024-05-09

**Authors:** Faith S. Reyes, Heather M. White, Kent A. Weigel, Jennifer M. C. Van Os

**Affiliations:** Department of Animal and Dairy Sciences, University of Wisconsin – Madison, Madison, WI, United States

**Keywords:** behavioral plasticity, dairy cattle, overstocking, residual feed intake, social dynamics

## Abstract

**Introduction:**

High feed bunk stocking densities can differentially impact individual dairy cows’ competitive behaviors, feeding patterns, and feed efficiency. Our objective was to manipulate feed bunk stocking densities to evaluate intra-individual behavioral consistency across stocking densities and quantify associations with feed efficiency and production.

**Methods:**

Thirty-two primiparous (130.7 ± 29.0 days in milk, DIM) and 32 multiparous (111.3 ± 38.3 DIM) lactating Holstein cows were housed with 32 roughage intake control (RIC) bins. Each cow was assigned to share 8 bins with others of the same parity and similar body weight (16 cows/block; 2:1 feed bunk stocking density except during tests). Competition and feeding patterns were evaluated via video and RIC data, respectively, at 3 stocking densities (1:1, 2:1, 4:1 cows/bin) during 1-h tests (2 tests/stocking density; 6 tests/cow) following 2 h feed deprivation. Residual feed intake (RFI) was calculated across the 45-d study as the difference between observed and predicted dry matter intake (DMI) after accounting for known energy sinks. Linear mixed models were used to evaluate the overall impact of test stocking density on competition and feeding patterns. To evaluate intra-individual consistency between stocking densities, individual stability statistic (ISS) scores were computed. Correlational relationships were determined between RFI and ISS scores.

**Results and dicsussion:**

Cows displayed the most competitive behaviors at 2:1 stocking density (*p* < 0.0001) but experienced the highest rate of contacts per minute of eating time at 4:1 (1:1 vs. 2:1 vs. 4:1: 0.09 vs. 0.95 vs. 1.60 contacts/min; *p* < 0.0001). Feeding patterns were modulated as stocking density increased; eating rate increased (0.16 vs. 0.18 vs. 0.22 kg/min; *p* < 0.001) as eating time (40.3 vs. 28.2 vs. 14.6 min; *p* < 0.001) and DMI decreased (6.3 vs. 5.1 vs. 3.0 kg; *p* < 0.001). As stocking density doubled, individuals remained consistent (*p* = 0.018) in time spent near others actively eating and tended to remain consistent in competition behavior and feeding patterns (0.053 ≤ *p* ≤ 0.094). Between 2:1 and 4:1, cows with higher DMI and milk production were more consistent in first-visit DMI and duration. Feed efficiency was not associated with behavioral consistency across the tests (*p* ≥ 0.14). Nonetheless, feed bunk stocking density has behavioral implications which should be considered in nutritional management decisions.

## 1 Introduction

Competition for resources, specifically feed, often occurs between cows on commercial dairy farms. Overcrowding dairy cows in free-stall barns at stocking densities greater than 1 cow per resting stall or headlock at the feed bunk (i.e., >100% capacity) may be utilized by dairy producers to reduce operating expenses or limit construction costs when making facility updates (1). A survey of North American dairy farms found that feed bunk stocking densities ranged from 58 to 228%, with an average of 142% in the northeastern United States (2). In the United States, on average, 67.9% of producers provided a feed bunk space allowance of less than 0.6 m, the industry recommendation (3). Stocking density at the feed bunk and its nutritional and welfare implications remain common topics for discussion in today’s dairy industry (4).

Increasing feed bunk stocking density has been shown to increase competitive behavior and alter feeding patterns of dairy cows. As stocking density at the feed bunk increases, cows are involved in more displacements at the bunk (5–7), spend more time standing in the feeding area (8), spend less time feeding (9), and eat at a faster rate (10). These changes in feeding patterns may impact feed efficiency, as faster eating rates have been associated with lower feed efficiency (11–13). In previous studies at a 2:1 stocking density, those cows involved in more direct competition at the feed bunk tended to be less feed efficient, dependent upon parity and group composition dynamics (13, 14).

Parity is often used as a proxy for dominance. However, we previously reported high individual variation, within parity, in cows’ latency to first visit the feed bunk after fresh feed delivery and in several other metrics of individual cows’ competitive success at the feed bunk; these patterns highlight that other factors contribute to social dynamics (13). Investigating individual characteristics that may contribute to this variation could provide insight into strategies cows utilize in competitive feed bunk environments. Under varying stocking densities, individual cows may either maintain or adjust their strategies, resulting in behavioral consistency or plasticity (15), respectively. For example, lactating cows displayed consistency in aggressor (individual initiating the interaction) behaviors but were not consistent in recipient (individual receiving the interaction) behaviors when provided different competitive feeding space allowances [0.6 vs. 0.3 m/cow; (16)]. To our knowledge, no other studies have explored the intra-individual consistency or plasticity of competitive behavior and feeding patterns in dairy cattle under varying feed bunk stocking densities, nor the relationship between individual behavioral consistency and feed efficiency. Depending on the environment and the individual, behavioral consistency or plasticity may be a potentially advantageous strategy. Behavioral plasticity has been measured using reaction norms to evaluate the behavioral response of an individual over an environmental gradient, which provides insight into how inter-individual variation interacts with the change in environment (15).

To address the knowledge gap in the literature on individual cows’ strategies across feed bunk stocking densities, we sought to manipulate stocking density in short-term testing scenarios by doubling the number of cows per feeding space (i.e., 1:1, 2:1, and 4:1). Our main objective was to evaluate intra-individual behavioral consistency or plasticity in competitive behaviors and feeding patterns among lactating dairy cows under different stocking densities and to characterize the associations between behavioral consistency, feed efficiency, and production. Relatively high stocking densities may have critical behavioral and feeding-pattern implications to consider when selecting management strategies. Nonetheless, our short-term evaluation of artificially high stocking densities was a methodological choice to evaluate cows’ responses when doubling stocking density and was not intended to reflect realistic industry practice.

## 2 Materials and methods

### 2.1 Animals, housing, and treatments

The study was conducted from May to July 2022 at the University of Wisconsin – Madison (UW-Madison) Emmons Blaine Dairy Cattle Research Center in Arlington, WI. All procedures were approved by the Institutional Animal Care and Use Committee (protocol # 005658-R02-A01).

Thirty-two primiparous (130.7 ± 29.0 DIM) and 32 multiparous (111.3 ± 38.3 DIM) lactating Holstein dairy cows were housed in a pen (53.3 × 12.6 m) with 64 sand-bedded resting stalls and equipped with 32 roughage intake control (RIC) system bins (Hokofarms Insentec BV, Marknesse, the Netherlands), which recorded individual cow feed intakes continuously. Cows were milked twice daily at 0300 and 1500 h in a double-sided 16-cow parallel parlor and fed thrice daily at 0900, 1500, and 2100 h. Fresh feed was delivered during the morning feeding; additional feed mixed in the morning was added to the bins in the afternoon feed deliveries. The same TMR diet was fed to all cows. Diet composition and nutrient analysis are presented in Supplementary Table S1. Refusals were manually recorded daily and feeding amounts were adjusted by parity to ensure all cows were fed *ad libitum*. Water was provided *ad libitum* via 3 automatic water troughs.

Blocks were formed to control for known variation in social dynamics related to BW and parity (13, 17) and evaluate the responses at the level of the individual cow at varying stocking densities. Each cow was assigned to share 8 adjacent bins with cows of the same parity and similar body weight (8 bins/block; 16 cows/block, standard 2:1 stocking density for RIC bins) (18). Each block was based on the combination of parity [primiparous (PR) or multiparous (MU)] and body weight (BW) [low (LO) or high (HI)], resulting in 4 blocks of n = 16 cows each (PR-LO, PR-HI, MU-LO, and MU-HI). Within each parity, the median BW (primiparous: 598.7 kg, multiparous: 747.9 kg) was used as the threshold for the 2 BW categories. Due to health issues unrelated to stocking density treatments, 1 MU-LO cow was removed in wk. 3 of the experimental period, resulting in 15 cows in MU-LO for the remainder of the study. Cow demographics by parity and BW are summarized in Table 1.

**Table 1:** Descriptive statistics^1^ for mid-lactation Holstein cows by block^2^

Variable	Primiparous, low BW	Primiparous, high BW	Multiparous, low BW	Multiparous, high BW
Starting DIM	117 ± 29 (70, 159)	144 ± 23 (85, 175)	93 ± 35 (59, 171)	128 ± 34 (75, 176)
BW, kg	586 ± 26 (525, 624)	662 ± 26 (617, 719)	728 ± 34 (652, 761)	813 ± 45 (764, 919)
Daily ∆BW	0.5 ± 0.3 (0.1, 1.0)	0.6 ± 0.2 (0.2, 1.0)	0.3 ± 0.4 (−0.3, 1.0)	0.7 ± 0.3 (0.02, 1.3)
BCS	3.4 ± 0.2 (3.1, 3.9)	3.4 ± 0.2 (3.2, 3.8)	3.1 ± 0.2 (2.9, 3.4)	3.3 ± 0.3 (2.8, 3.9)
Total ∆BCS^3^	−0.03 ± 0.1 (−0.4, 0.3)	−0.1 ± 0.1 (−0.3, 0.1)	0.0 ± 0.1 (−0.3, 0.3)	0.01 ± 0.2 (−0.2, 0.5)
Lactation	1.0	1.0	3.4 ± 1.5 (2, 6)	4.4 ± 1.1 (2, 6)
Milk yield, kg/d	39.3 ± 5.2 (31.1, 48.3)	39.1 ± 4.7 (30.1, 45.6)	56.5 ± 7.6 (43.5, 69.6)	51.8 ± 10.9 (31.2, 68.2)
Milk energy output, Mcal/d	28.6 ± 2.9 (23.9, 34.4)	29.8 ± 3.5 (24.3, 34.8)	37.4 ± 4.3 (29.3, 42.6)	34.2 ± 5.3 (22.2, 40.3)
Residual feed intake^4^	−0.3 ± 1.0 (−2.4, 1.2)	0.3 ± 1.0 (−1.4, 2.1)	−0.1 ± 1.5 (−3.0, 2.0)	0.1 ± 2.2 (−3.8, 5.4)

^1^Mean ± SD with range in parentheses listed for each variable. ^2^Cows were assigned to blocks of 16 cows each by combination of parity (PR: primiparous or MU: multiparous) and BW (LO: low BW or HI: high BW); MU-LO had only 15 cows after removal due to illness unrelated to the study. ^3^Change over 42 d. ^4^Estimation of feed efficiency, calculated for each cow by regressing DMI on milk energy output, median DIM, metabolic BW, and change in BW, each nested within parity.

All MU cows had experience with the RIC system in previous lactations, whereas the RIC bins were novel to PR cows. All cows were trained to their assigned bins during a 1-wk period and exposed once (10-min/training session) to each of 3 competitive testing scenarios (1 cow:1 bin, 2:1, and 4:1) to be used during the experimental period, in a randomized order of exposure. Outside of the training sessions, cows had access to only their 8 assigned bins (standard 2:1 stocking density). Cows were considered trained once ≤30% of daily attempted bin visits were directed to non-assigned bins (mean ± SD: 18.3 ± 7.7%; range: 0.0–30.6%). Once training was complete, the experimental period lasted 45 d, which included the competitive testing periods in the first 4 wk.

### 2.2 Competitive tests

Competitive tests were performed for each block of cows under all three stocking densities (1 cow:1 bin, 2:1, and 4:1; Figure 1). Before each test, the cows were feed deprived for 2 h to standardize minimum time since last feeding and increase feeding motivation; resting stalls (1 stall/cow) were accessible and water was provided *ad libitum* during this period.

**Figure 1:**
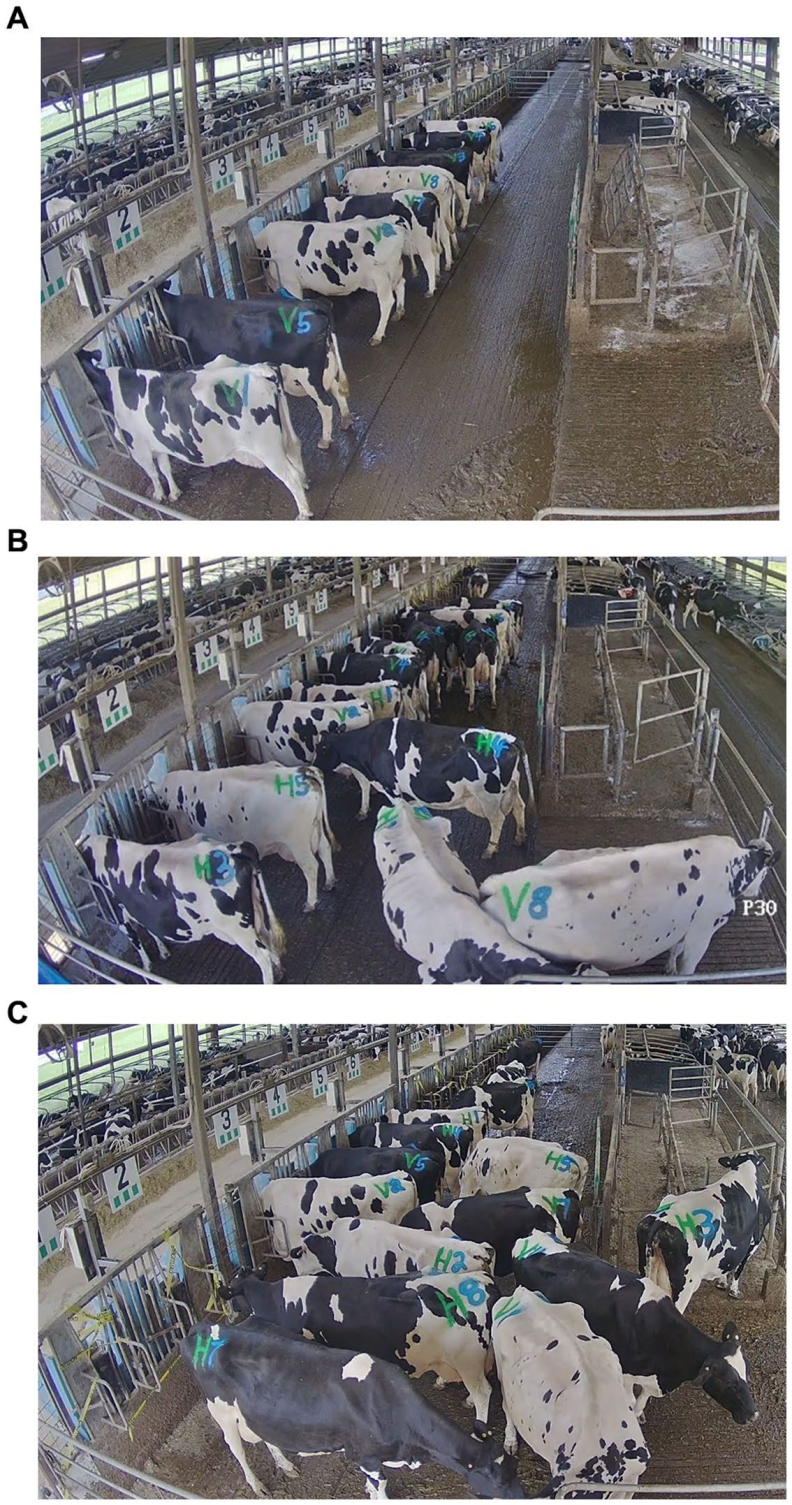
Screenshots from video recordings of 1-h tests of each feed bunk stocking density treatment: **(A)** 1:1 (8 cows: 8 bins), **(B)** 2:1 (16 cows: 8 bins), and **(C)** 4:1 (16 cows: 4 bins).

Two tests were performed with different groups of cows each day, immediately after fresh feed delivery at 0900 and 1500 h. For the 1:1 tests, blocks were randomly divided in half because only 8 bins were available; these subgroups remained consistent throughout training and testing at this stocking density. Test cows were limited to half of the feed bunk (16 bins) during each test (26.7 × 2.4 m, 64.1 m^2^ testing area). In the 1:1 and 2:1 tests, the 8 bins unassigned to test cows were empty, manually locked closed, and blocked with caution tape. In the 4:1 tests, only the 4 central assigned bins were accessible; the 2 normally assigned bins on each end were empty, manually locked for the test period, and blocked with caution tape to discourage attempts to access those bins.

Once fresh feed was delivered to bins accessible during the test, the test cows were moved from the stall area to the open half of the feed bunk. During tests, all non-test cows were locked to the stall side of the pen and did not have access to the feed bunk. Water access was provided *ad libitum* throughout the feed deprivation period and competition tests for all cows, whether tested or not. The test was conducted for 1 h, during which RIC data and continuous video were collected. Once each test was complete, all cows were provided free access to the entire pen.

Each group was tested twice (once each in the morning and afternoon on separate days) at each stocking density, with the order of exposure balanced among groups. For MU-LO, which was missing one cow, both 4:1 tests were performed with only 15 cows, and one 1:1 test was performed with a randomly chosen cow from the other subgroup to achieve 8 cows/8 bins.

### 2.3 Measures

#### 2.3.1 Competition behavior

**Figure 2:**
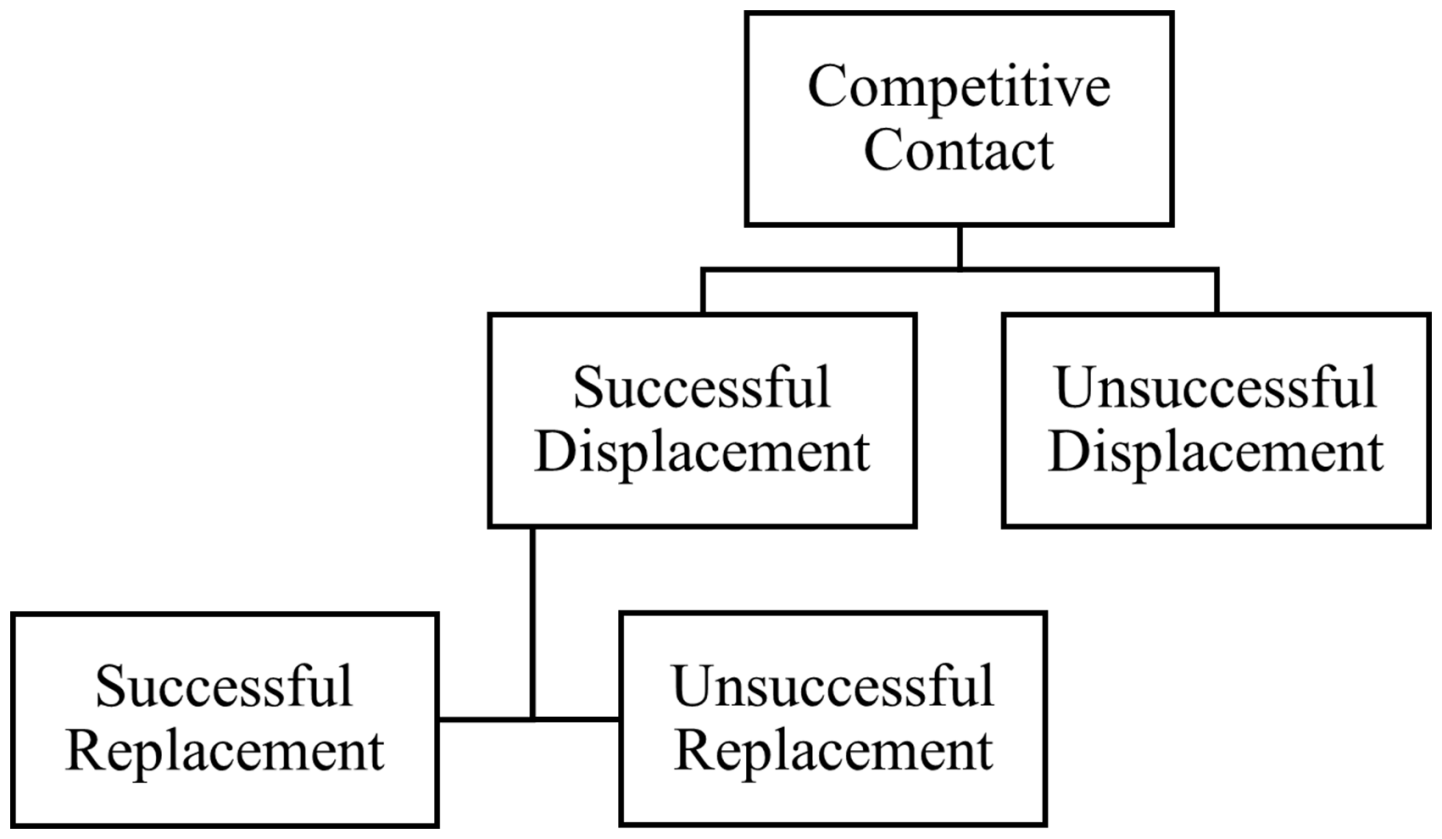
Flowchart of the behavior sequence used for observation of competitive interactions at the feed bunk between mid-lactation Holstein cows. For each behavior, an actor (cow initiating the event) and receiver (individual receiving the event) were recorded.

Continuous video for each 1-h test was recorded using 10 cameras (Platinum 4.0 MP Network Matrix IR Bullet Camera, CMIP9342W-28 M; LT Security Inc., Washington, NY) mounted at 3.7 m high, which were set to record with 2,688 × 1,520 resolution at 10 frames/s through a network video recorder (Platinum Enterprise Level 64 Channel NVR, LTN8964-8; LT Security Inc.). Each cow was marked with spray paint (Tell Tail, FIL Industries Limited, Mount Maunganui, New Zealand) for individual identification. Three trained observers coded the video recordings, observed using VSPlayer (Hikvision Digital Technology, Hangzhou, China), for competitive interactions using continuous sampling (defined in Table 2; sequence shown in Figure 2) and for behavioral inventories using instantaneous scan sampling in 5-min intervals [defined in Table 2; 1 body length (~2 m) was visually assessed compared to the width of 2.5 RIC bins for scale]. Inter-observer reliability was determined on a subsample of video that included all focal behaviors (scan sampling: 13 scans during a 1-h period with 16 cows/scan, 208 observations total; continuous sampling: 715 observations total during a 1-h period). Cohen’s kappa was calculated by subtracting the probability of random agreement from the observed agreement, divided by the probability of random agreement subtracted from one (PROC FREQ; 9.4, SAS Institute). The resulting value yields Cohen’s kappa, with values closer to 1.0 indicating greater agreement between observers. Our calculated Cohen’s kappa ranged from 0.85 to 1.0 for instantaneous scan sampling indicating “almost perfect” agreement and ranged from 0.61 to 1.0 for continuous sampling, indicating “substantial” to “almost perfect” agreement (19) for all behaviors except unsuccessful displacement attempts (κ = 0.60, indicating “moderate” agreement).

**Table 2:** Ethogram used for observing feed bunk interactions and proportions calculated from counts of competition behavior at the feed bunk during 1-h tests at different feed bunk stocking densities.

Variable	Definition	Ratio	Actor/Receiver index
Continuous sampling			
Competitive contact	Actor makes physical contact with receiver eating at a bin. The event stops when the actor ceases physical contact.	Rate of received contacts: Received competitive contacts/total eating time	Competitive (actor) index: Initiated competitive contacts/sum of initiated and received competitive contacts
Successful displacement	The physical contact performed by the actor results in the receiver backing out of the bin completely, so that her head is no longer through the metal bars of the feed bunk and/or the bin’s gate closes.	Successful displacement (actor) ratio: Initiated successful displacements/total initiated competitive contacts	Displacement (actor) index: Initiated successful displacements/sum of initiated and received successful displacements
Successful replacement	The successful displacement results in the actor entering the bin and the gate opens to allow for her to begin to eat.	Successful replacement (actor) ratio: Initiated successful replacements/total initiated competitive contacts. Successful displacement to replacement (actor) ratio: Initiated successful replacements/total initiated successful displacements	Replacement (actor) index: Initiated successful replacements/total initiated and received successful replacements
Unsuccessful displacement	After the physical contact performed by the actor, the receiver continues to eat at the bin.	Displacement resistance (receiver) ratio: Received unsuccessful displacements/total received competitive contacts	
Unsuccessful replacement	The successful displacement does not result in the actor accessing and eating from the same bin.		
Instantaneous scan sampling			
Eating	Head in the feed bin with the gate open	–	–
Loitering	Standing ≤1 body length (~2 m) away from an eating cow	–	–
Not loitering	Standing >1 body length away from an eating cow	–	–

#### 2.3.2 Feeding behavior and dry matter intake

Individual TMR ingredients were collected weekly and dried by forced air oven (Isotemp Oven, Fisher Scientific, Waltham, MA) at 55°C for 48 h (concentrates in triplicate, forages in quadruplicate), composited by week and analyzed by a commercial laboratory (Dairyland Labs, Inc., Arcadia, WI) as described previously (20). In brief, samples were analyzed for DM determined by National Forage Testing Association method 2.1.4, crude protein (method 990.03), neutral detergent fiber (method 2002.04), acid detergent fiber (method 973.18), lignin (method 973.18), ether extract (method 920.39), ash (method 942.05), water-soluble carbohydrates, and starch. Total mixed ration nutrient profile was determined by averaging the nutrient profile of actual daily ingredient inclusion in the diet on a DM basis. The weekly ingredient samples were dried at 105°C for 24 h (in duplicate) to convert feed intake to a DM basis.

The feed intake and bin visit details (time of day, duration, bin location) during the tests were recorded automatically by the RIC system, which collected data 24 h/d; day-level DMI data were used for calculating RFI. A visit was defined as a single event that occurred when a cow entered an assigned bin and associated RIC data were recorded. Other variables derived from RIC data were latency (min) to first access a feed bin after test start, number of visits/h, DMI/h, eating rate (kg/min), and total eating time (min/h, regardless of intake). Due to the competitive nature of the tests, not all individuals accessed a feed bin during each 1-h test. In this instance, the latency to first access a feed bin after test start and eating rate were considered undefined and were not included in the analysis (19 total data points, 17 from 4:1 tests and 2 from 2:1 tests). Dry matter intake and duration during the first successful visit to the feed bunk were calculated, along with summed eating time within the first 30 min after test start; 30 min was selected based on the average length of a meal (21). Daily intakes (kg as fed) on test dates were compared with intakes on days cows were not tested (Supplementary Table S2) to ensure that testing did not impact daily intakes, which were used when calculating RFI.

#### 2.3.3 Milk yield and components

Milk yields were recorded in DairyCOMP 305 (Valley Ag Software, Spencer, MA) and summarized as kg/d for each cow. Milk samples from 4 consecutive milkings/wk. were collected and preserved with 2-bromo-2-nitropro-pane-1,3-diol (Advanced Instruments Inc., Norwood, MA) and analyzed at a commercial laboratory (AgSource, Menomonie, WI) for milk composition (fat, protein, lactose, and milk urea nitrogen) and SCC.

#### 2.3.4 Residual feed intake

Residual feed intake was calculated as a measure of feed efficiency for each cow (greater value indicates less efficient) by regressing DMI on milk energy output, median DIM, metabolic BW, and change in BW, each nested within parity. All values were summarized as an average across the 45-d experimental period for each cow. Milk energy output (kg/d) was calculated as [9.29 × milk fat (kg)] + [5.63 × true protein (kg)] + [3.95 × lactose (kg)] (22). Body weight was recorded before morning feed delivery 3 d/wk. during wk. 1, 4, and 7 of the experimental period using a calibrated stationary scale (EW6, Tru-Test Limited). Metabolic BW (kg) was calculated as BW^0.75^. The daily change in BW was calculated using the LINEST function in Microsoft Excel to create a simple linear regression of all 9 BW values. Body condition score (reported descriptively in Table 1; not used in RFI) was assessed on 1 d in wk. 1, 4, and 7 in conjunction with BW by 2 trained observers using the 5-point scale (Dairy Body Condition Score Chart, Elanco Animal Health) at increments of 0.25.

### 2.4 Statistical analysis

#### 2.4.1 Missing and excluded data

Video for a portion (last 25 min) of one 1:1 test for PR-HI cows was not recorded due to equipment malfunction.

#### 2.4.2 Statistical models

All response variables were analyzed using R programming language (R version 4.2.1) or SAS software (9.4, SAS Institute). Cow was the experimental unit. Residuals were assessed visually using graphs and numerically using the Shapiro–Wilk test for normality as necessary.

For our main objective, which was to evaluate individual behavioral consistency, we used individual stability statistic (ISS) scores to evaluate consistency of competition behavior and feeding patterns across pairs of stocking densities at the feed bunk. For competition behavior, only competitive contacts (initiated, received, and total) were included in the analysis to represent the start of the sequence of competitive events; all feeding pattern variables were included in the analysis. For each cow, ISS scores were calculated for each variable at each pair of stocking density treatments, as described in R Core Team (23):

$$\mathrm{IS}S_{xy}=\frac{1-{\left(z_{x}-z_{y}\right)}^{2}}{2}$$

where z refers to the z-score of a given variable within the x and y stocking density treatments. Because ISS is calculated pairwise between situations and we were interested in consistency across the 3 tests as stocking densities doubled, we computed ISS between the 1:1 vs. 2:1 and the 2:1 vs. 4:1 pairs of tests. The calculated ISS scores were skewed and thus further transformed to achieve approximate normal distributions (24, 25). A higher ISS score is indicative of less change (greater consistency) between two stocking density treatments. To evaluate consistency among all 3 testing scenarios, Pearson correlations were performed between ISS_1:1,2:1_ and ISS_2:1,4:1_ to evaluate consistency as stocking density changed when doubling the number of cows per bin (from 1:1 to 2:1 and 2:1 to 4:1). Additionally, Pearson correlations were used to evaluate relationships between test consistency (separately for ISS_1:1,2:1_ and ISS_2:1,4:1_) and RFI, DMI, and milk production. Significance was defined at a threshold of *p* < 0.05 and tendencies as *p* ≤ 0.10.

For completeness in reporting, we also evaluated the overall impact of stocking density on competition and feeding patterns using linear models (*lmer* package; 23). Non-normal continuous variables (latency to first visit a bin, first visit DMI and duration, number of bin visits, and eating rate) were log_n_ transformed to improve normality. Generalized linear mixed models (PROC GLIMMIX, SAS) were used to evaluate effects on count-based competition variables and proportions (competition indexes and ratios) using a negative binomial or Poisson distribution, based on model fit. Latency to the first bin visit was analyzed using a gamma distribution. These models included fixed effects of stocking density treatment, block, and their interaction, as well as a random effect of cow. One PR-HI cow did not access a RIC bin during each of the 4:1 tests; therefore, variables related to feeding behavior and successful feed bunk access were not included in the analysis for that individual. All values are reported as least-squares means.

## 3 Results

### 3.1 Behavioral consistency and feed efficiency

**Figure 3:**
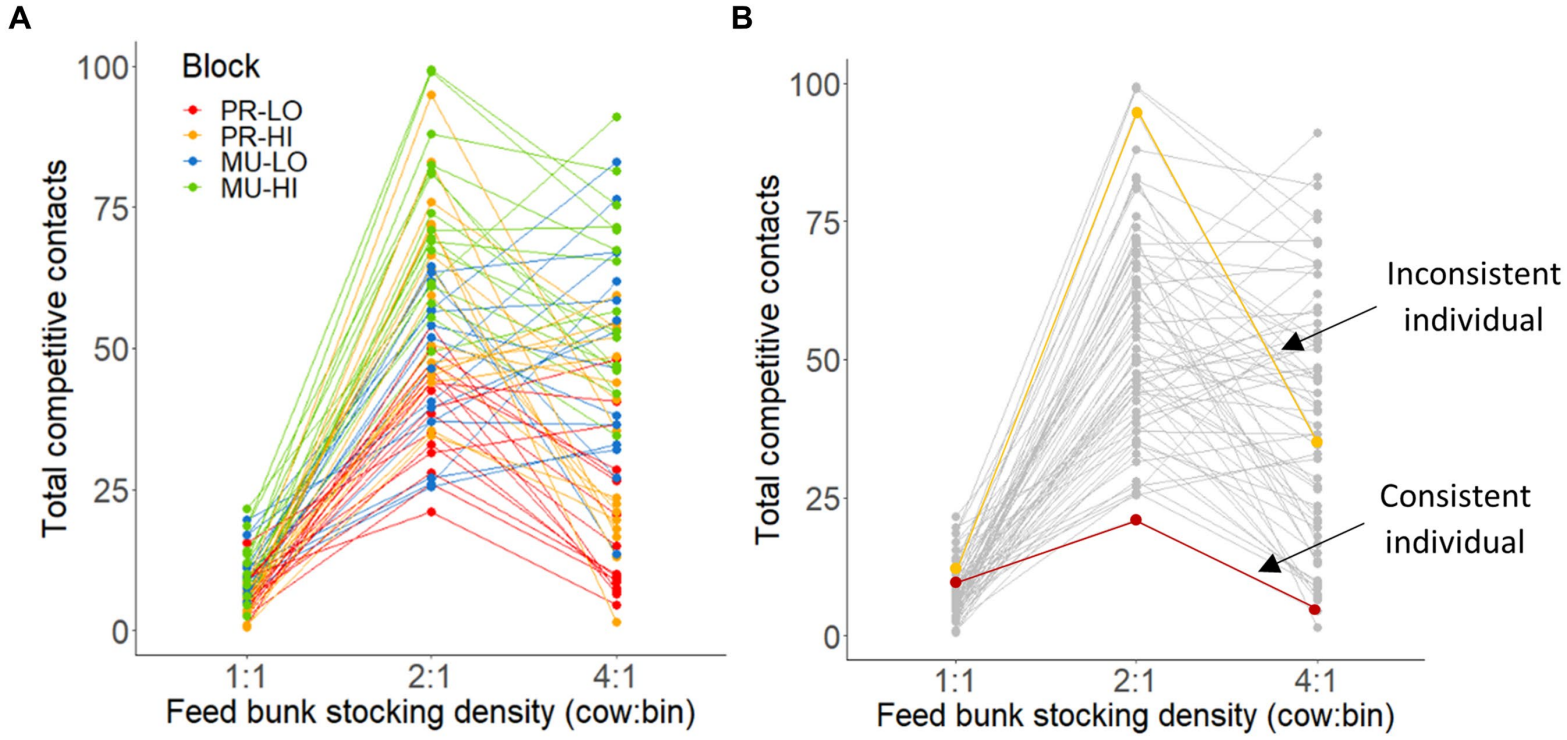
**(A)** Descriptive behavioral reaction norm showing individual mid-lactation Holstein cows’ total competitive contacts when feed bunk stocking density doubled (1 cow: 1 bin, 2:1, or 4:1) in 1-h tests (averaged between 2 tests per stocking density). Cows were assigned to blocks of 16 cows each by combination of parity (PR: primiparous or MU: multiparous) and body weight (LO, low bodyweight or HI, high bodyweight). **(B)** The same plot is shown with 2 specific individuals colored as examples of being relatively consistent (red line) vs. inconsistent (yellow line).

An example of a descriptive behavioral reaction norm plot for total competitive contacts is shown in Figure 3, with an example for interpreting ISS scores for relatively consistent vs. inconsistent individuals across stocking densities; similar plots for all variables are shown in Supplementary Figure S3.

The intra-individual behavioral consistencies for all variables across different stocking densities (i.e., correlations between ISS_1:1,2:1_ and ISS_2:1,4:1_) are summarized in Table 3. When cows were more consistent between the 1:1 and 2:1 stocking densities in total competitive contacts, latency to the first bin visit, number of visits, or total eating time, they also tended to remain more consistent for those variables between the 2:1 and 4:1 tests (R range: 0.22–0.24, *p* ≤ 0.094). In addition, when cows were more consistent between the 1:1 vs. 2:1 stocking densities for the proportion of time spent loitering within 1 body length of an actively eating cow, they were also more consistent between the 2:1 and 4:1 stocking densities (*R* = 0.30, *p* = 0.018). No other variables showed consistency across the stocking density comparisons (R range = 0.06–0.30, *p* ≥ 0.13).

**Table 3:** Pearson correlations between individual stability statistic (ISS) scores^1^ of behavioral and feeding patterns calculated between different pairs of stocking densities^2^ at the feed bunk, applied during 1-h tests, in mid-lactation Holstein cows.

Variable	R-value	*p*-value
Total competitive contacts^3^	0.24	0.063
Competitive contacts (actor)^3^	0.14	0.26
Competitive contacts (receiver)^3^	0.10	0.44
Prop. of time spent loitering^4^	0.30	0.018
Prop. of time spent not loitering^4^	0.06	0.59
Latency to first visit a bin, min	0.24	0.061
DMI of first visit, kg	0.09	0.50
First visit duration, min	0.15	0.25
Eating time within the first 30 min, min	0.10	0.45
Number of bin visits	0.24	0.053
DMI, kg	0.19	0.13
Eating rate, kg/min	0.10	0.44
Total eating time, min	0.22	0.094

^1^Individual stability statistic (ISS) scores calculated as a measure of behavioral consistency between two different stocking densities; higher values (not shown) indicate more consistency. ^2^ISS scores were calculated between 1:1 and 2:1 stocking densities (1 vs. 2 cows/bin) and between 2:1 and 4:1 (2 vs. 4 cows/bin). Correlations were computed between ISS_1:1,2:1_ and ISS_2:1,4:1_. ^3^Physical contact between two individuals with one cow initiating the contact (actor) and the other receiving the contact while eating at the feed bunk (receiver); total represents the sum between initiated and received competitive contacts. ^4^Proportion of time spent performing each behavior within 1 h; averaged between two 1-h tests for each stocking density. Behaviors included loitering (standing within 1 body length of an eating cow), and not loitering (standing more than 1 body length away from an eating cow).

### 3.2 ISS and feed efficiency

**Figure 4:**
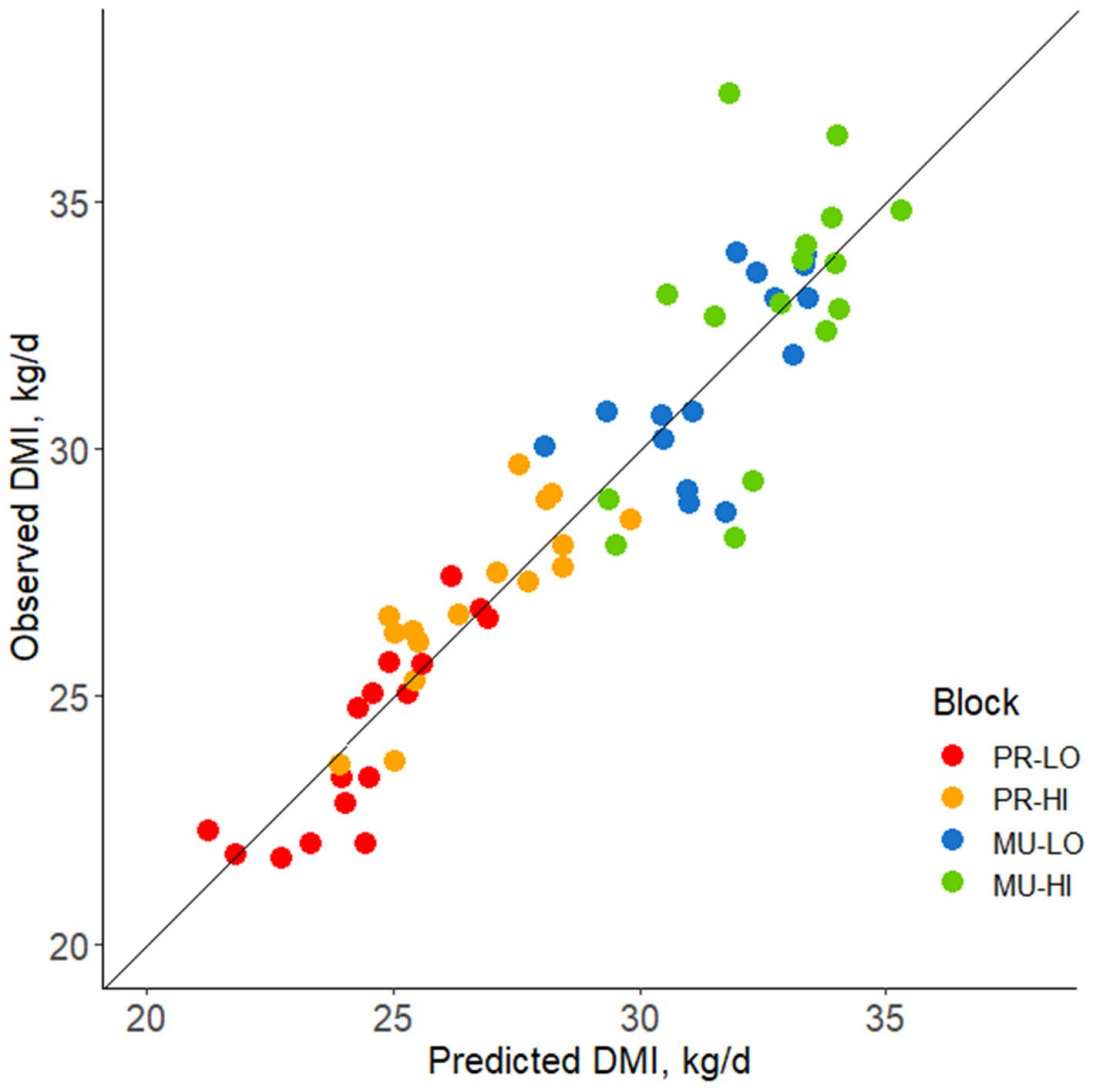
Observed vs. predicted DMI plotted for each block [based on parity (PR, primiparous; MU, multiparous) and body weight (LO, low bodyweight; HI, high bodyweight)] for mid-lactation Holstein cows. Data points above the line of unity represent cows consuming more feed than predicted, associated with a positive residual feed intake (RFI) value and lesser feed efficiency. Data points below the line of unity represent cows consuming less feed than predicted, associated with a negative RFI and greater feed efficiency.

Residual feed intake values based on the comparison between observed and predicted DMI are shown in Figure 4. Relationships between individual consistency across the stocking density tests and feed efficiency are summarized in Table 4. Feed efficiency was not associated with consistency across stocking density comparisons for any test variable (R range = −0.19 to 0.12, *p* ≥ 0.14).

**Table 4:** Pearson correlations^1^ between residual feed intake^2^ during the 45-d study and individual stability statistic (ISS) scores^3^ for responses during 1-h tests across different stocking densities^4^ at the feed bunk in mid-lactation Holstein cows.

Variable	ISS_1:1,2:1_	ISS_2:1,4:1_
Total competitive contacts^5^	0.12 (0.35)	−0.08 (0.56)
Competitive contacts (actor)^5^	0.03 (0.83)	−0.01 (0.93)
Competitive contacts (receiver)^5^	−0.0001 (0.99)	−0.18 (0.16)
Prop. of time spent loitering^6^	−0.05 (0.71)	−0.03 (0.82)
Prop. of time spent not loitering^6^	−0.14 (0.27)	−0.03 (0.82)
Latency to first visit a bin, min	−0.11 (0.38)	−0.02 (0.87)
DMI of first bin visit, kg	−0.12 (0.36)	0.02 (0.89)
First visit duration, min	−0.08 (0.55)	0.10 (0.44)
Eating time within the first 30 min, min	−0.05 (0.86)	−0.03 (0.80)
Number of bin visits	−0.05 (0.67)	0.02 (0.90)
DMI, kg	−0.05 (0.71)	−0.19 (0.14)
Eating rate, kg	0.10 (0.44)	−0.10 (0.45)
Total eating time, min	−0.07 (0.60)	0.06 (0.67)

^1^R-value is given, with *P*-value in parentheses. ^2^Estimation of feed efficiency, calculated for each cow by regressing DMI on milk energy output, median DIM, metabolic BW, and change in BW, each nested within parity. ^3^Individual stability statistic (ISS) scores calculated as a measure of behavioral consistency across two different stocking densities; higher values (not shown) indicate more consistency. ^4^ISS scores were calculated between 1:1 and 2:1 stocking densities (1 vs. 2 cows/bin) and between 2:1 and 4:1 (2 vs. 4 cows/bin). Correlations were computed between ISS_1:1,2:1_ and ISS_2:1,4:1_. ^5^Physical contact between two individuals with one cow initiating the contact (actor) and the other receiving the contact while eating at the feed bunk (receiver); total represents the sum between initiated and received competitive contacts. ^6^Proportion of time spent performing each behavior within 1 h; averaged between two 1-h tests for each stocking density. Behaviors included loitering (standing within 1 body length of an eating cow), and not loitering (standing more than 1 body length away from an eating cow).

### 3.3 ISS and production metrics

Relationships between individual consistency across the stocking density tests and average daily DMI and milk production are summarized in Tables 5, 6, respectively. Cows with higher DMI throughout the trial were more consistent between the 2:1 and 4:1 stocking densities in latency to the first bin visit and first visit duration (R range: 0.26–0.39, *p* ≤ 0.041). These individuals with greater daily DMI also tended to remain consistent between the 2:1 and 4:1 stocking densities in the DMI of the first bin visit (*R* = 0.24, *p* = 0.057). In addition, cows consuming higher daily DMI showed less consistency between the 2:1 and 4:1 stocking densities in DMI during the 1-h test (*R* = −0.27, *p* = 0.032) and tended to show less consistency in received competitive contacts (*R* = −0.21, *p* = 0.096). Higher milk producing cows were more consistent in first visit duration (*R* = 0.35, *p* = 0.005) and tended to be more consistent in DMI of the first bin visit (*R* = 0.24, *p* = 0.061) between the 2:1 and 4:1 stocking densities. Milk production and DMI were not associated with consistency across stocking density comparisons for any other test variables (R range = −0.19 to 0.19, *p* ≥ 0.13).

**Table 5:** Pearson correlations^1^ between average daily dry matter intake^2^ during the 45-d study and individual stability statistic (ISS) scores^3^ for responses during 1-h tests across different stocking densities^4^ at the feed bunk in mid-lactation Holstein cows.

Variable	ISS_1:1,2:1_	ISS_2:1,4:1_
Total competitive contacts^5^	0.08 (0.55)	−0.14 (0.28)
Competitive contacts (actor)^5^	−0.10 (0.42)	−0.02 (0.86)
Competitive contacts (receiver)^5^	−0.10 (0.43)	−0.21 (0.096)
Prop. of time spent loitering^6^	−0.07 (0.58)	0.13 (0.31)
Prop. of time spent not loitering^6^	0.08 (0.51)	0.09 (0.48)
Latency to first visit a bin, min	−0.07 (0.60)	0.26 (0.041)
DMI of first bin visit, kg	−0.01 (0.95)	0.24 (0.057)
First visit duration, min	0.07 (0.61)	0.39 (0.002)
Eating time within the first 30 min, min	−0.01 (0.95)	0.12 (0.33)
Number of bin visits	−0.12 (0.37)	−0.19 (0.14)
DMI, kg	−0.17 (0.18)	−0.27 (0.032)
Eating rate, kg	−0.09 (0.47)	−0.14 (0.27)
Total eating time, min	0.10 (0.43)	0.18 (0.17)

^1^R-value is given, with P-value in parentheses. ^2^Average daily dry matter intake (DMI) across the entire 45-d study. ^3^Individual stability statistic (ISS) scores calculated as a measure of behavioral consistency across two different stocking densities; higher values (not shown) indicate more consistency. ^4^ISS scores were calculated between 1:1 and 2:1 stocking densities (1 vs. 2 cows/bin) and between 2:1 and 4:1 (2 vs. 4 cows/bin). Correlations were computed between ISS_1:1,2:1_ and ISS_2:1,4:1_. ^5^Physical contact between two individuals with one cow initiating the contact (actor) and the other receiving the contact while eating at the feed bunk (receiver); total represents the sum between initiated and received competitive contacts. ^6^Proportion of time spent performing each behavior within 1 h; averaged between two 1-h tests for each stocking density. Behaviors included loitering (standing within 1 body length of an eating cow), and not loitering (standing more than 1 body length away from an eating cow).

**Table 6:** Pearson correlations^1^ between average milk production^2^ during the 45-d study and individual stability statistic (ISS) scores^3^ for responses during 1-h tests across different stocking densities^4^ at the feed bunk in mid-lactation Holstein cows.

Variable	ISS_1:1,2:1_	ISS_2:1,4:1_
Total competitive contacts^5^	0.13 (0.32)	−0.14 (0.27)
Competitive contacts (actor)^5^	0.01 (0.94)	−0.18 (0.16)
Competitive contacts (receiver)^5^	−0.11 (0.38)	−0.12 (0.37)
Prop. of time spent loitering^6^	−0.06 (0.64)	0.11 (0.38)
Prop. of time spent not loitering^6^	0.18 (0.16)	−0.01 (0.93)
Latency to first visit a bin, min	−0.07 (0.60)	0.17 (0.18)
DMI of first bin visit, kg	0.13 (0.31)	0.24 (0.061)
First visit duration, min	0.10 (0.41)	0.35 (0.005)
Eating time within the first 30 min, min	0.01 (0.96)	0.07 (0.57)
Number of bin visits	−0.18 (0.17)	−0.06 (0.66)
DMI, kg	−0.04 (0.76)	−0.08 (0.54)
Eating rate, kg	−0.02 (0.89)	−0.13 (0.30)
Total eating time, min	0.14 (0.28)	0.19 (0.13)

^1^R-value is given, with P-value in parentheses. ^2^Average daily milk production across the entire 45-d study. ^3^Individual stability statistic (ISS) scores calculated as a measure of behavioral consistency across two different stocking densities; higher values (not shown) indicate more consistency. ^4^ISS scores were calculated between 1:1 and 2:1 stocking densities (1 vs. 2 cows/bin) and between 2:1 and 4:1 (2 vs. 4 cows/bin). Correlations were computed between ISS_1:1,2:1_ and ISS_2:1,4:1_. ^5^Physical contact between two individuals with one cow initiating the contact (actor) and the other receiving the contact while eating at the feed bunk (receiver); total represents the sum between initiated and received competitive contacts. ^6^Proportion of time spent performing each behavior within 1 h; averaged between two 1-h tests for each stocking density. Behaviors included loitering (standing within 1 body length of an eating cow), and not loitering (standing more than 1 body length away from an eating cow).

### 3.4 Stocking density main effects

Cows were involved in the greatest number of competitive behavior events (initiated, received, and total) at the intermediate (2:1) stocking density, compared with 1:1 and 4:1, for all events in the sequence (*p* < 0.001) (Table 7). Competition ratios and indexes did not differ across stocking densities. The received contact ratio was highest at the 4:1 stocking density compared to 1:1 and 2:1 (*p* < 0.0001) (Table 7). During one-hour testing periods, cows spent the lowest proportion of time loitering within 1 body length of cows eating when tested at the 1:1 stocking density, compared with 2:1 and 4:1 (*p* < 0.001) (Table 7). In addition, cows spent the greatest proportion of time standing more than 1 body length away from cows eating when tested at the 4:1 stocking density (*p* < 0.001), whereas 1:1 and 2:1 did not differ (Table 7).

**Table 7:** Competition behavior^1^ at the feed bunk recorded during 1-h tests at different stocking densities for mid-lactation Holstein cows, blocked by parity and body weight.

Variable	1 cow:1 bin	2 cows:1 bin	4 cows:1 bin	*p-*value		
				Trt	Block	Trt*Block
Event counts^2^						
Competitive contacts (actor)	3.9 (3.3,4.5) ^c^	26.9 (24.1,30.0) ^a^	18.8 (16.7, 21.0) ^b^	< 0.001	< 0.001	< 0.001
Competitive contacts (receiver)	3.8 (3.1,4.5) ^c^	26.5 (23.3, 30.2) ^a^	18.7 (16.4,21.4) ^b^	< 0.001	< 0.001	< 0.001
Total competitive contacts	7.6 (6.7,8.7) ^c^	53.6 (48.5,59.3) ^a^	37.4 (33.7,41.4) ^b^	< 0.001	< 0.001	< 0.001
Successful displacements (actor)	1.1 (0.9,1.4) ^c^	5.4 (4.7,6.1) ^a^	4.0 (3.5,4.6) ^b^	< 0.001	< 0.001	< 0.001
Successful displacements (receiver)	1.1 (0.8,1.4) ^c^	5.3 (4.6,6.1) ^a^	3.9 (3.4,4.6) ^b^	< 0.001	< 0.001	< 0.001
Unsuccessful displacements (receiver)	2.6 (2.1,3.2) ^c^	20.6 (17.9,23.9) ^a^	14.7 (12.6,17.1) ^b^	< 0.001	0.001	< 0.001
Successful replacements (actor)	0.8 (0.6,1.1) ^c^	3.7 (3.2,4.3) ^a^	2.4 (2.0,2.8) ^b^	< 0.001	< 0.001	0.028
Successful replacements (receiver)	0.8 (0.6,1.0) ^c^	3.6 (3.2,4.1) ^a^	2.4 (2.1,2.9) ^b^	< 0.001	< 0.001	0.014
Ratios						
Rate of received contacts^3^	0.09 (0.04,0.20) ^c^	0.95 (0.78,1.16) ^b^	1.60 (1.41,1.84) ^a^	< 0.001	< 0.21	0.73
Successful displacement ratio^4^	0.32 (0.21,0.50)	0.24 (0.14,0.40)	0.22 (0.13,0.38)	0.53	0.71	0.76
Displacement resistance ratio^5^	0.64 (0.47,0.88)	0.76 (0.56,1.00)	0.71 (0.53,0.95)	0.78	0.79	0.96
Successful replacement ratio^6^	0.24 (0.14,0.40)	0.18 (0.10,0.32)	0.16 (0.09,0.30)	0.49	0.61	0.72
Displacement to replacement ratio^7^	0.76 (0.55,1.04)	0.69 (0.51,0.93)	0.63 (0.45,0.87)	0.70	0.70	0.99
Indexes						
Competitive index^8^	0.49 (0.35,0.70)	0.50 (0.35,0.71)	0.55 (0.39,0.77)	0.88	0.99	1.0
Displacement index^9^	0.49 (0.34,0.71)	0.51 (0.36,0.72)	0.50 (0.35,0.72)	0.99	0.99	0.99
Replacement index^10^	0.52 (0.45,0.59)	0.49 (0.41,0.57)	0.49 (0.42,0.58)	0.75	0.88	0.98
Behavior Inventory^11^						
Eating^12^	0.68 ± 0.02	0.47 ± 0.02	0.25 ± 0.02	–	–	–
Loitering	0.15 ± 0.02 ^c^	0.34 ± 0.02 ^b^	0.40 ± 0.02 ^a^	<0.001	0.10	0.12
Not-loitering	0.18 ± 0.02 ^b^	0.19 ± 0.02 ^b^	0.35 ± 0.02 ^a^	<0.001	0.29	0.02

^abc^Superscripts indicate treatment main effects for each variable. ^1^Back-transformed means and 95% confidence intervals reported from a natural logarithm-based negative binomial distribution, unless otherwise specified. ^2^Averaged between two 1-h tests at each stocking density. ^3^Received competitive contacts/total eating time (min). ^4^Initiated successful displacements/initiated competitive contacts. ^5^Received unsuccessful displacements/received competitive contacts. ^6^Initiated successful replacements/initiated competitive contacts. ^7^Initiated successful replacements/initiated successful displacements. ^8^Initiated competitive contacts/total competitive contacts initiated and received. ^9^Initiated successful displacements/total successful displacements initiated and received. ^10^Initiated successful replacements/total successful replacements initiated and received. ^11^Proportion of time spent performing each behavior within 1 h; averaged between two 1-h tests for each stocking density. Behaviors included eating (head in the feed bin), loitering (standing within 1 body length of an eating cow), and not loitering (standing more than 1 body length away from a cow eating).^12^Not included in analysis, as eating time is already represented in total eating time from electronic bin data.

As stocking density increased, latency to the first bin visit and eating rate increased, whereas DMI, eating time within the first 30 min, and total eating time decreased (*p* < 0.001) (Table 8). At the 4:1 stocking density, compared with the lower stocking densities, cows’ first bin visits were the shortest, and the least amount of feed was consumed; they also visited the bunk less frequently during those 1-h tests, while feeding patterns did not differ between 1:1 and 2:1 (*p* < 0.001) (Table 8). For completeness, stocking density × block effects are reported in Tables 7, 8; however, these interactions were not of primary interest to the objectives of this study and are not further discussed.

**Table 8:** Comparison of feeding patterns at different stocking densities, averaged between two 1-h tests per stocking density, for mid-lactation Holstein cows, blocked by parity and body weight.

Variable	1 cow:1 bin	2 cows:1 bin	4 cows:1 bin	*p-*value		
				Trt	Block	Trt*Block
Latency to first visit a bin, min^1^	2.8 (2.1, 3.7) ^c^	5.3 (4.0, 7.1) ^b^	14.7 (10.9, 19.7) ^a^	< 0.001	0.58	0.78
DMI of first visit, kg	2.1 (1.9, 2.4) ^a^	1.0 (0.7, 1.3) ^b^	1.1 (0.9, 1.4) ^b^	< 0.001	0.46	0.007
First visit duration, min	12.0 (10.6, 13.3) ^a^	5.2 (3.9, 6.6) ^b^	5.7 (4.4, 7.1) ^b^	< 0.001	< 0.001	0.008
Eating time within first 30 min, min	23.3 ± 0.7 ^a^	13.2 ± 0.7 ^b^	6.8 ± 0.7 ^c^	< 0.001	0.98	0.99
Number of bin visits^1^	5.8 (5.3, 6.4) ^a^	6.8 (6.2, 7.5) ^a^	3.9 (3.6, 4.3) ^b^	< 0.001	< 0.001	<0.001
DMI, kg	6.3 ± 0.2 ^a^	5.1 ± 0.2 ^b^	3.0 ± 0.2 ^c^	< 0.001	< 0.001	0.89
Eating rate, kg/min^1^	0.16 (0.15, 0.16) ^c^	0.18 (0.17, 0.19) ^b^	0.22 (0.21, 0.23) ^a^	< 0.001	< 0.001	<0.001
Total eating time, min	40.3 ± 1.2 ^a^	28.2 ± 1.2 ^b^	14.6 ± 1.2 ^c^	< 0.001	0.66	0.34

^1^Back-transformed means and 95% CI from a natural logarithm-based distribution. ^abc^Superscripts within a row indicate significant treatment main effects, *p* ≤ 0.05.

## 4 Discussion

The purpose of this study was to measure competitive behaviors and feeding patterns under varying feed bunk stocking densities in order to evaluate intra-individual behavioral consistency across those testing scenarios and associations with feed efficiency and production. As stocking density doubled (1:1 vs. 2:1 and 2:1 vs. 4:1), cows showed intra-individual consistency in the proportion of time spent waiting at the feed bunk (i.e., within 1 body length of an actively eating cow), and they tended to show consistency in competitive behavior and feeding patterns. Behavioral consistency across the testing scenarios was associated with dry matter intake and milk yield during the 45-d experiment, but not with feed efficiency. On average, cows showed the most involvement in competitive interactions at the intermediate 2:1 stocking density. In addition, on average, they modulated all feeding patterns as stocking density increased, presumably to adjust for the lack of opportunity to access the feed bunk; in particular, eating rate increased as eating time and DMI decreased.

### 4.1 Individual behavioral consistency

To our knowledge, this is the second study to evaluate intra-individual behavioral consistency of dairy cows in competitive behaviors as stocking density increases, but the first to evaluate more than two stocking densities and to utilize ISS scores. A previous study (16) reported, using correlations, that lactating cows were consistent at the individual level in the proportion of initiated contacts vs. non-contact behaviors (threats and blocking) as open-rail feeding space was halved from the standard 0.6–0.3 m per cow, but not in interactions as the receiver. In the present study, we found cows who were more consistent between the 1:1 and 2:1 tests for total competitive contacts tended to be more consistent between the 2:1 and 4:1 tests. Furthermore, we found a weak, positive (although not statistically significant) correlation in consistency in received competitive contacts across the stocking densities. Variation in competition involvement at the feed bunk by role (actor, receiver) represents an area for future research to continue to explore behavioral strategies and consistency.

In addition to evaluating direct competitive interactions, we observed proximity of cows to others who were actively eating during the tests. When cows were within 1 body length of another actively eating cow, this could indicate their potential or motivation to gain access to the bunk, including through direct competitive behavior. Cows who were more consistent between the 1:1 vs. 2:1 stocking densities (between which feed bin stocking density doubled and total space per cow halved) for the proportion of time spent time near an actively eating cow were more consistent between the 2:1 and 4:1 stocking densities (between which feed bin stocking density doubled, but space per cow in the testing area remained constant). These findings may suggest that cows utilize similar strategies to attempt to obtain access to the feed bunk, regardless of the feed bunk stocking density or space available near the feed bunk.

Our study is the first to evaluate the intra-individual behavioral consistency of feeding patterns across multiple stocking densities. Cows who were more consistent between the 1:1 vs. 2:1 stocking densities for latency to first visit a bin, frequency of visits, or total eating time tended to be more consistent between the 2:1 and 4:1 stocking densities in these variables. These findings further suggest that cows appear to have individual strategies that persist across different stocking densities which they utilize to attempt to gain access to feed.

Importantly, it should be noted that intra-individual consistency coexists with inter-individual (within-group) variability (26, 27). That is, individuals may remain consistent for certain behaviors across different environments, but those responses may vary between individuals within and across environments, thus yielding inter-individual variation. These individual differences in biological responses demonstrate the importance of understanding individual-level responses in addition to group-level patterns.

One limitation of the consistency metric we used is that it can only compare two environments at a time. To compare across the 3 stocking densities in our study, we correlated two pairwise ISS scores; this approach has not been previously used in other studies. Behavioral plasticity has also been analyzed in wildlife contexts using a behavioral reaction norm approach with random regression (15), or character state models can be applied to discrete environments (28), but both of these alternative methods require a vastly larger number of subsamples for accurate estimation of intra- and inter-individual variance in each context.

#### 4.1.1 Individual behavioral consistency and feed efficiency

We did not find novel associations between individual behavioral consistency across the stocking density tests and feed efficiency. In our previous work at a 2:1 stocking density, less efficient cows tended to have higher DMI in the first bin visit after fresh feed delivery (14). Additionally, increased eating rate has been associated with lower feed efficiency in a previous study (12) and in our other work in a similar environment with 2:1 stocking density (11, 13). Faster rates of feed consumption on a meal-basis can reduce rumen pH (29), which may impact digestion and nutrient utilization, and thus feed efficiency. These relationships between behavior and feed efficiency highlight the potential for common connections. We initially sought to characterize the behavioral attributes of a feed efficient cow, hypothesizing that perhaps cows utilize specific behaviors to gain successful access to the feed bunk in competitive environments, and that individuals may be consistent across stocking densities in their individual strategies, which may be connected to feed efficiency. Our study did not observe any associations with RFI and behavioral consistency, but the potential interrelationships between feed efficiency and behavior in various environments highlights an interesting area for future research.

#### 4.1.2 Individual behavioral consistency and production metrics

We identified novel associations between individual behavioral consistency across stocking densities and production metrics involving DMI and milk production. Between the higher stocking density tests (2:1 and 4:1), cows who had higher daily DMI and milk production showed, or tended to show, more consistency in first visit DMI and duration. This may suggest that consistency in feeding strategy at the first bunk visit after fresh feed delivery, particularly in a highly competitive environment, may impact feed access and potentially production. Greater DMI and milk production has been reported to be associated with specific daily feeding behaviors, including feeding time and meal frequency, in high-producing cows (28). Characterization of the first bunk visit after fresh feed delivery has not been a regular focus of past research, but our group’s previous work at a 2:1 stocking density highlighted interesting relationships with first visit factors and feed efficiency (13, 14). Furthermore, in the present study when comparing the two higher stocking densities, we found that cows with greater daily DMI were more consistent in latency to the first bunk visit during the test, which further supports the importance of the strategy during the first visit. Interestingly, cows with greater daily DMI showed less consistency between the 2:1 and 4:1 stocking densities in the DMI during the 1 h test period. Perhaps, regardless of first visit strategies, cows who showed behavioral plasticity and were able to adjust to the situation of the 1 h test were also individuals who consumed the most feed on average throughout the 45 d study.

Associations between intra-individual behavioral consistency across stocking densities and feed efficiency were based on 1 h tests. We did this to focus on peak feeding times, when social dynamics and dominance play major roles in gaining access to fresh feed (9); however, cows have been shown to adjust their strategies and patterns throughout the day (30). Longer exposure to higher stocking densities may result in different competitive behavior and feeding patterns. In addition, providing access to the entire pen, including resting stalls, could influence the patterns observed, as cows are not only highly motivated to access fresh feed (30), but also resting areas (31). Further evaluation of intra-individual behavioral consistency and plasticity across varying stocking densities (including those between 1:1 and 2:1, more reflective of farm settings) for longer periods of time, as well as their relationships with feed efficiency, daily DMI, and milk production, are potentially interesting topics for future research. Advancing our understanding of individual behavioral strategies has the potential to assist in on-farm decisions regarding stocking densities, and optimizing these strategies may lead to improved nutritional management on dairy farms.

### 4.2 Overall stocking density implications

Although the main objective of this study was to evaluate individual consistency in behavior and feeding patterns across different stocking densities at the feed bunk, we also report average differences between the testing scenarios for completeness and to allow comparison with previous literature. From an applied perspective, our study and previous work show that high stocking densities have critical behavioral and feeding-pattern implications which should be considered when selecting management strategies on farm. Nonetheless, this does not imply our tests were intended to reflect a realistic application of high stocking densities in a practical setting. It is also important to note that the differences between our three testing scenarios likely reflect multiple factors which varied between stocking densities, including both group size and space availability, based on the design of each test stocking density. Another caveat is that we tested cows in blocks based on parity (primiparous vs. multiparous) and bodyweight (high vs. low). This was done deliberately to minimize the effects of these blocking factors within each test group, as previous research demonstrated that these factors can affect both competition and feeding patterns (32–34). Because our main objective was to evaluate intra-individual consistency across stocking densities, concurrently with inter-individual differences in response patterns and consistency, mixing parities or bodyweight extremes within a testing group could have masked our ability to evaluate individual patterns. Some block effects and stocking density treatment by block interactions were detected, confirming that indeed, parity and bodyweight affect cows’ competitive and feeding responses. A limitation of our study design is that we had only one block per parity and bodyweight combination; future studies could strengthen the design with greater replication by having multiple test groups for each of these combinations.

#### 4.2.1 Competition events

Higher stocking densities at the feed bunk have been previously shown to impact competition, although the direction of the effect is variable across studies. In our study using 1-h tests, we found that, overall, cows were involved in the highest level of competition behavior at the intermediate 2:1 stocking density, with slightly less at 4:1 but greater than at 1:1, when all cows could eat simultaneously. This nonlinear pattern could be explained by the reduced bunk space in the 4:1 test, and thus opportunity to compete for bunk access (i.e., displace another cow eating at the bunk). Alternatively, individuals may have changed their strategy at a 4:1 stocking density to avoid direct competition, rather than attempting to compete for access to feed.

Our finding of greater competition at 2:1 vs. 1:1 stocking density is consistent with another study that compared stocking densities between 1 and 2 lactating cows per feeding space over longer observation periods. This study reported that displacements increased at higher stocking densities (7). However, another study found the opposite pattern, where cows exhibited fewer aggressive interactions and displacements when feeding space per cow decreased from 0.6 to 0.4 m (16), which could be partly explained by differences in feed bunk design. In a study in which stocking density increased at both the feed bunk and resting stalls, the number of bunk displacements did not vary (8). This may have been due to competing motivations to lie down after milking rather than consume fresh feed when both resources were limited (31), which was not the case in the current study.

Fewer studies have evaluated feed bunk stocking densities greater than 2 cows per feeding space. Consistent with our findings, one older study showed that displacements increased with 4:1 vs. 1:1 cows per electronic feeding bin (10), but the study did not have an intermediate treatment. Another study did not detect differences in the number of successful replacements per day [extrapolated from RIC data of Huzzey et al. (35)] as stocking density increased from 1:1 to 3:2 and 3:1 cows per bin (36); however, they did not evaluate displacement or replacement attempts, which require video analysis. More studies are needed to evaluate whether our nonlinear response pattern in direct competition across stocking densities would be replicated in other settings. During our 1-h tests, cows could not access the resting stalls, potentially resulting in more direct interactions due to limited space (8 m^2^/cow in the 1:1 test; 4 m^2^/cow in the 2:1 and 4:1 tests) and resources.

#### 4.2.2 Competition ratios and indexes

When considering the amount of time spent eating at the feed bunk, cows received competitive contacts at the highest rates in the 4:1 stocking density test. Although the number of competitive events were fewer in the 4:1 compared to 2:1 stocking density, more competitive contacts were received within shorter periods of eating time. We speculate there may have been behavioral feedback cycles wherein certain individuals may have attempted to avoid direct competition, perhaps in part because those who successfully accessed the feed bunk were subject to increased competitive pressure at higher stocking densities.

The other competition behavior ratios and indexes in our study were not impacted by stocking density. A previous study likewise found no difference in displacement and aggression indices (similar to our study’s displacement and competitive indices, respectively) when cows had 0.6 vs. 0.3 m of feeding space (16). In addition, in the current study, all indexes and ratios showed high inter-individual variation, as we observed in our previous work at 2:1 RIC bin stocking density (13, 14) and as observed by others who reported displacement indexes with 0.34 m of linear post-and-rail feed bunk space per cow (37) and 1:1 or 2:1 RIC bin stocking density (38). On a numerical basis, all actor-based ratios, regardless of stocking density, were lower than our previous studies in the same pen [both 2:1 stocking density; (13, 14)], meaning cows were less successful when attempting to gain access to feed. Conversely, the displacement resistance ratio was numerically higher than in our previous studies, indicating that cows were “standing their ground” and resisting displacement by not leaving the bunk. These numerical observations could, in part, be explained by cows learning from repeated exposure to the 4:1 condition (during both the training period and the 2 tests) that bunk access was sometimes scarce in the current study. In previous studies involving strict criteria for discrimination learning, lactating cows learned in less than 90 cumulative minutes across sessions to discriminate between feedstuffs based on color cues (39), and calves learned to locate the presence of a milk reward in less than 40 cumulative minutes (40), although lactating cows in another study required longer timeframes when learning to discriminate colors and shapes (41). In our study, we assumed that cows could learn to identify differences in bunk access during the training and testing sessions, which cumulatively comprised 130 min per treatment. In addition to learning to recognize the differences in stocking density among the tests, the cows may have learned that the test conditions were of limited duration. As in some previous studies by others (9), one of the stocking density treatments (2:1) was also utilized for general housing outside of testing periods for the remainder of the day. Therefore, cows may have anticipated the return to a 2:1 stocking density after the tests, which could have impacted strategies to engage in or avoid competition to gain access to feed as well as their feeding patterns during the tests.

#### 4.2.3 Feeding patterns and proximity to cows actively eating

When 1 bin was available per cow during the 1-h test, cows spent approximately 40 min eating. This was reduced to 28 min in the 2:1 test, which is nearly identical to the average meal durations of 27 to 29 min that we reported previously at the same stocking rate in this pen (13, 14). At the 4:1 stocking density, the number of available bins was halved, as was eating time (a 64% decrease relative to the 1:1 scenario). These patterns were as predicted and were consistent with the general observations in previous studies that eating time decreased as a function of space per cow or with stocking densities >100%, either at the 24-h level (5, 9) or during peak times after fresh feed delivery (5, 42).

When cows were not eating, we recorded their proximity to other cows who were actively eating from a bin as a possible reflection of their motivation to compete for bunk access, in addition to quantifying direct competition. At the 1:1 stocking density (8 m^2^/cow in the testing area), when cows were not eating, they spent roughly equal proportions of time either less or greater than 1 body length away from cows actively eating. Time spent within 1 body length of cows actively eating was 2.3- and 2.7-fold greater, respectively, in the 2:1 and 4:1 tests (4 m^2^/cow in the testing area in both of those tests). Time spent more than 1 body length away was similar between the 1:1 and 2:1 tests, when all 8 bins were active, and this proportion nearly doubled in the 4:1 test, when only 4 bins were available. These patterns could suggest cows were attempting to avoid direct competition in the 4:1 test, in which we observed less direct competition than in the 2:1 scenario. However, this could also be a limitation of the space available near cows actively eating when only 4 could eat at a time, as our behavioral definition was based on proximity to cows eating rather than proximity to the feed bunk. In a previous study, as feeding space decreased from 0.8 to 0.2 m/cow, cows spent 78% more time standing in the feed alleys (9).

Consistent with previous literature, as stocking density increased and eating time decreased, cows ate more rapidly (10, 36), likely in an attempt to compensate for reduced access to the feed bunk. Average eating rate increased by 12.5% between the 1:1 and 2:1 tests and by another 22% in the 4:1 test (a total increase of 37.5% compared to 1:1). This strategy was partially successful given that DMI during the test decreased by 19% between the 1:1 and 2:1 tests and by another 41% in the 4:1 test (a total decrease of 52% compared to 1:1) but was not halved as stocking density doubled. A previous study evaluating DMI during peak feeding time likewise found that DMI decreased as stocking density increased from 89 to 129% (42). However, stocking density did not affect eating rate or DMI at the 24-h level in that study or two others that used RIC bins [1 cow:1 bin vs. 2:1, (40); 1:1 vs. 3:2 vs. 3:1, (36)]. Likewise, in our study, cows seemed able to compensate and maintain daily DMI. We observed that DMI did not differ significantly between days that cows were or were not tested, with the exception of PR-LO cows (difference of 1.7 ± 0.7 kg). However, it is worth noting that the previous studies maintained the assigned stocking density for at least an entire 24-h period, whereas in our study a 2:1 stocking density at the feed bunk was used after the testing periods each day.

In terms of visit and meal patterns, cows did not differ in the frequency of bunk visits between the 1:1 and 2:1 tests, consistent with previous studies that evaluated these stocking densities (38, 43), but the number of visits was reduced in the 4:1 test. Previous work comparing 1:1 and 2:1 stocking densities also found that cows exhibited less frequent, longer meals (i.e., related visits within a certain duration) in the latter (44). In our study, we did not characterize meal variables during the 1-h tests, because some meals may have been interrupted by the end of the test, when all cows in the pen were allowed access to the feed bunk area. Instead, we characterized patterns when each cow first successfully gained access to the feed bunk during the test. We found that, as the number of cows per bin doubled between the 1:1 vs. 2:1 tests, the average latency to first access a bin also doubled. However, as the stocking density doubled again to 4:1, latency increased by 2.8-fold (a total 5.3-fold increase relative to 1:1), which could be due to reduced physical space around the 4 bins reducing accessibility or to delayed approach as a strategy to avoid competition. Interestingly, once cows first gained access to a bin, duration of the first visit was reduced by more than half in the 2:1 vs. 1:1 test but was not further reduced in the 4:1 test; DMI during that first visit followed the same pattern.

In addition to first visit patterns, we calculated the amount of time each individual spent eating within the first 30 min of the 1-h test. We based this duration on the length of an average meal (21). This measure was used to evaluate variation in those individuals able to access feed quickly after the test started (i.e., during the first potential meal) compared to those who were unable to gain access or waited to access the feed bunk. The amount of time cows spent eating within the first 30 min essentially halved each time stocking density doubled, decreasing by 43% when stocking density doubled from 1:1 to 2:1 and by another 48% as stocking density doubled once more from 2:1 to 4:1. These patterns provide additional support to suggest some cows may have been attempting to avoid direct competition in the 4:1 test, during which cows spent on average more time more than 1 body length away from a cow eating than in the 2:1 test.

## 5 Conclusion

As stocking density in the tests doubled, individual cows remained consistent for time spent within 1 body length of actively eating cows (i.e., waiting at the bunk), and tended to remain consistent for competition behavior and feeding patterns. At higher stocking densities, cows with higher DMI and milk production were more consistent in first-visit DMI and duration. However, intra-individual consistency across the stocking density tests was not associated with feed efficiency during the 45-day study. Overall, feed bunk stocking density impacts competition behavior and feeding patterns in lactating dairy cows. In this study, competitive interactions were greatest at the intermediate 2:1 stocking density, while the rate of received competitive interactions per minute of eating time was highest at the 4:1 stocking density. On average, cows modulated their feeding patterns to adjust to limited access to the feed bunk; eating rate increased, perhaps to partially compensate for decreased eating time and DMI. It is important to note that the inferences of our findings are limited to improving our understanding of the effects of feed bunk stocking density on responses, particularly individual consistency across contexts, during a short-term test. The stocking densities we tested were not intended to be extrapolated to practical farm settings. Nonetheless, our findings reiterate the important behavioral implications of high stocking densities at the feed bunk, while also highlighting the behavioral complexities of intra-individual consistency across different testing stocking densities.

## Data availability statement

The raw data supporting the conclusions of this article will be made available by the authors, without undue reservation.

## Ethics statement

The animal study was approved by the University of Wisconsin-Madison Institutional Animal Care and Use Committee. The study was conducted in accordance with the local legislation and institutional requirements.

## Author contributions

FR: Data curation, Formal analysis, Investigation, Methodology, Software, Validation, Visualization, Writing – original draft. HW: Conceptualization, Funding acquisition, Methodology, Resources, Supervision, Writing – review & editing. KW: Conceptualization, Funding acquisition, Methodology, Resources, Supervision, Writing – review & editing. JO: Conceptualization, Funding acquisition, Methodology, Project administration, Resources, Supervision, Writing – review & editing.
