# Supplementary material for: Behavioral consistency of competitive behaviors and feeding patterns in lactating dairy cows across stocking densities at the feed bunk
Source: Front Vet Sci. 2024 May 9;11:1302573. doi: 10.3389/fvets.2024.1302573 (PMC11112419; doi:10.3389/fvets.2024.1302573)
Supplement: Supplementary file 1 [file Data_Sheet_1.docx]

Supplementary Material

Behavioral consistency of competitive behaviors and feeding patterns in lactating dairy cows across stocking densities at the feed bunk

F. S. Reyes, H. M. White, K. A. Weigel, J. M. C. Van Os^*^

*** Correspondence:** J.M.C. Van Os: jvanos@wisc.edu

# Supplementary Figures and Tables

**Supplementary Table S1.** Calculated ingredient composition and nutrient analysis of the diet fed to primiparous and multiparous mid-lactation Holstein cows

| Item, % of DM | Mean |
| --- | --- |
| Ingredient composition |  |
| Alfalfa haylage | 22.42 ± 1.10 |
| Corn silage | 27.42 ± 1.45 |
| Distillers grain | 2.26 ± 0.09 |
| Cottonseed | 4.86 ± 0.08 |
| Ground corn grain | 13.81 ± 0.27 |
| Protein mineral mix^1^ | 29.22 ± 0.51 |
| Nutrient analysis |  |
| DM, % as fed | 51.77 |
| OM | 92.32 |
| CP | 18.10 |
| NDF | 26.17 |
| ADF | 21.97 |
| Lignin | 4.09 |
| NFC | 44.65 |
| Starch | 27.27 |
| Fat | 4.49 |
| NE_L_ 3x^2^ | 1.61 |

^1^Protein mineral mix was formulated on an as-fed basis to contain fine ground corn (26.33%), soy hull pellet (18.09%), 46% CP soybean meal (17.22%), canola meal (15.60%), SoyPlus (11.60%, Landus Cooperative), calcium carbonate (4.62%), sodium bicarbonate (2.50%), trace mineral salt (1.25%), magnesium oxide (0.82%), urea (0.62%), potassium carbonate (0.30%), Celmanax Dry (0.30%, Arm & Hammer), grease (0.25%), Smartamine M (0.20%, Adisseo), DynaMate (0.15%, The Mosaic Company), and Fortress LG (0.16%, VitaPlus).

^2^Estimated from the NASEM (2022) equations to calculate NE_L_ at 3× maintenance.

**Supplementary Table S2.** Daily intake comparison^1^ between test day vs. non-test day intakes (on an as-fed basis) for mid-lactation Holstein cows.

|  | Intake Type | |  | |  |
| --- | --- | --- | --- | --- | --- |
| Block^2^ | Test Day Intake | Non-Test Day Intake | | *P*-value | |
| PR-LO |  |  | |  | |
| All cows | 47.3 ± 1.1 | 46.9 ± 0.9 | | 0.36 | |
| Subgroup A | 49.1 ± 1.6 | 46.9 ± 1.6 | | 0.0002 | |
| Subgroup B | 45.6 ± 1.2 | 46.8 ± 1.0 | | 0.026 | |
| PR-HI |  |  | |  | |
| All cows | 51.8 ± 1.0 | 52.6 ± 0.9 | | 0.36 | |
| Subgroup A | 51.9 ± 1.5 | 51.6 ± 1.4 | | 0.49 | |
| Subgroup B | 51.7 ± 1.3 | 53.8 ± 1.2 | | 0.18 | |
| MU-LO |  |  | |  | |
| All cows | 61.9 ± 1.2 | 61.8 ± 1.1 | | 0.89 | |
| Subgroup A | 63.9 ± 1.4 | 62.4 ± 1.5 | | 0.33 | |
| Subgroup B | 59.9 ± 1.9 | 61.1 ± 1.6 | | 0.25 | |
| MU-HI |  |  | |  | |
| All cows | 65.3 ± 1.8 | 64.9 ± 1.4 | | 0.71 | |
| Subgroup A | 64.1 ± 2.2 | 65.9 ± 1.9 | | 0.18 | |
| Subgroup B | 66.5 ± 2.9 | 64.0 ± 2.1 | | 0.16 | |

^1^Means and SE reported from paired t-tests.

^2^Cows were assigned to blocks of 16 cows each by combination of parity (PR: primiparous or MU: multiparous) and BW (LO: low BW or HI: high BW); MU-LO had only 15 cows after removal due to illness unrelated to the study. Within block, cows were randomly assigned to two subgroups of 8 cows each for 1:1 tests. Each test day was included for the subgroup averages.


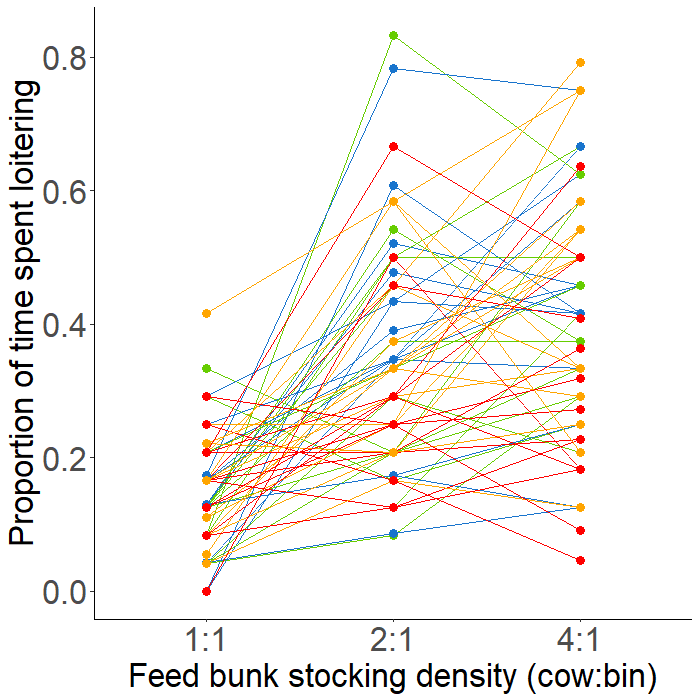

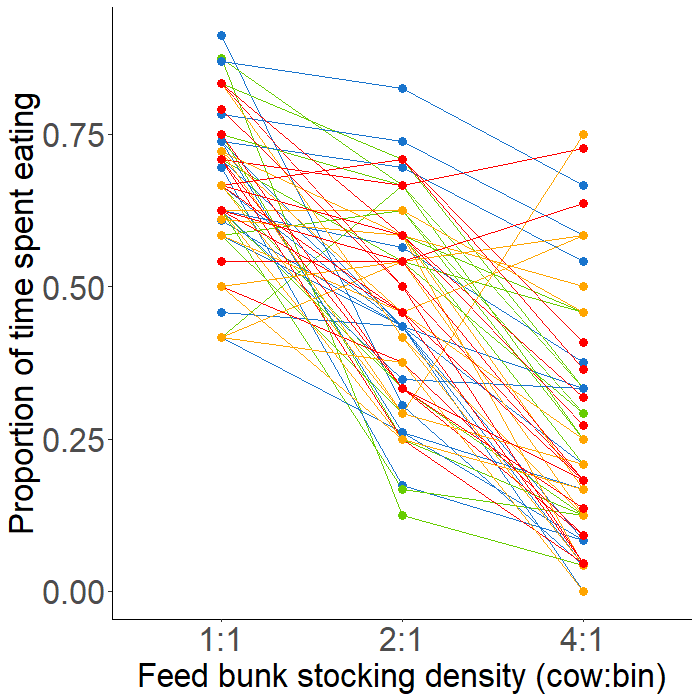

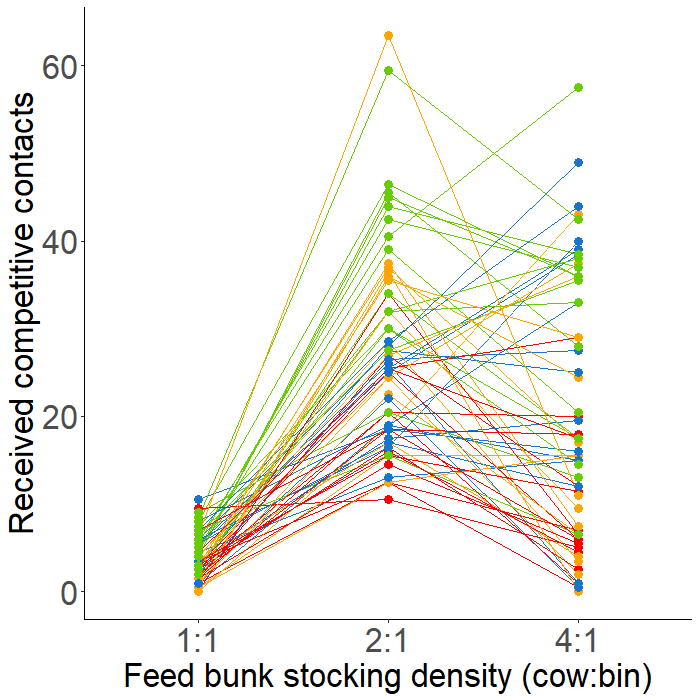

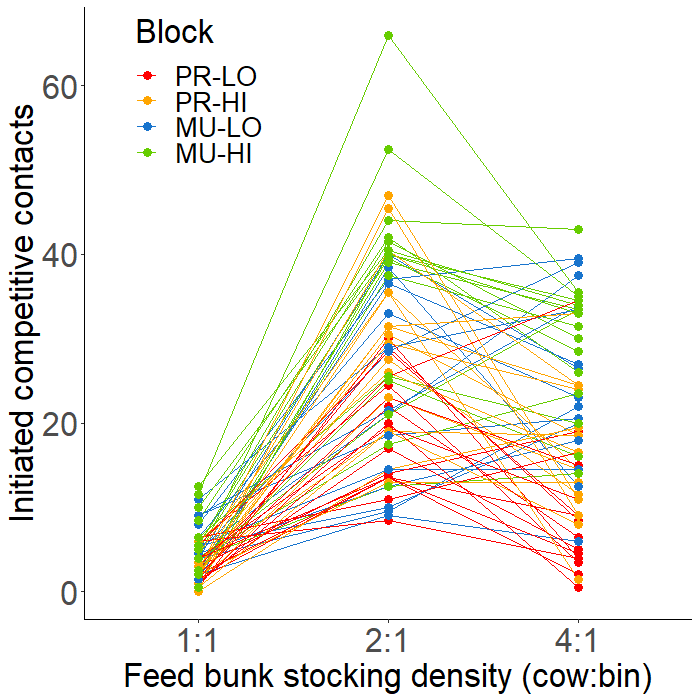
**Supplementary Figure S3.** Descriptive behavioral reaction norms showing individual mid-lactation Holstein cows’ competition behavior (A-E) and feeding pattern (F-M) responses to changes in feed bunk stocking density (1 cow: 1 bin, 2:1, or 4:1) in 1-h tests (averaged between 2 tests per stocking density). Cows were assigned to blocks of 16 cows each by combination of parity (PR: primiparous or MU: multiparous) and body weight (LO: low bodyweight or HI: high bodyweight); MU-LO had only 15 cows after removal due to illness unrelated to the study.

**(D)**

**(B)**

**(C)**

**(A)**

**(H)**

**(G)**

**(F)**

**(E)**


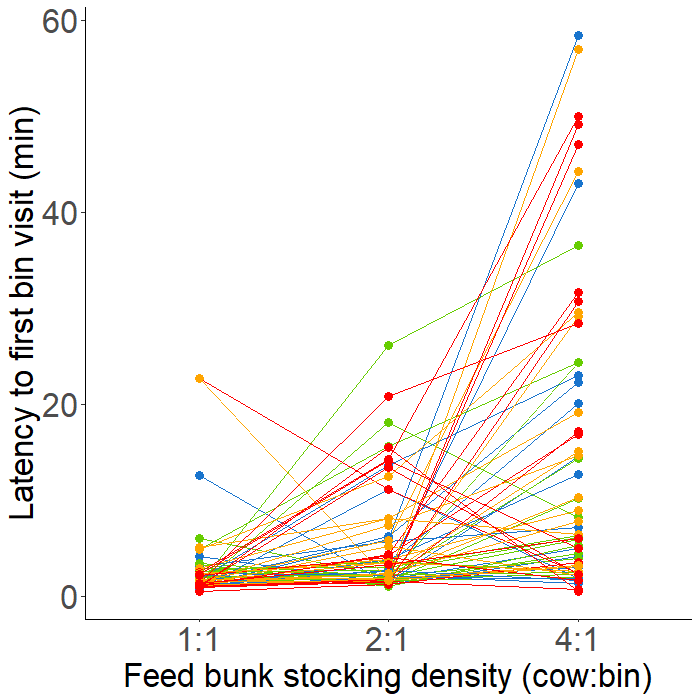

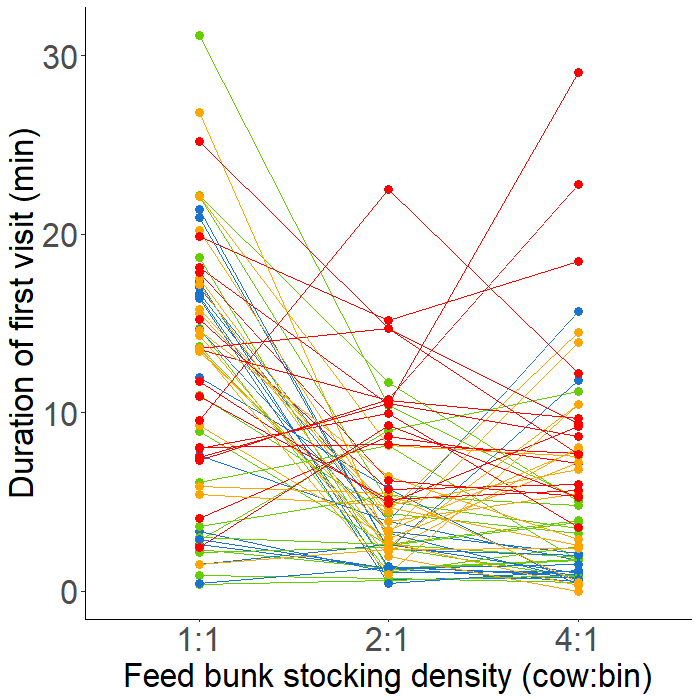

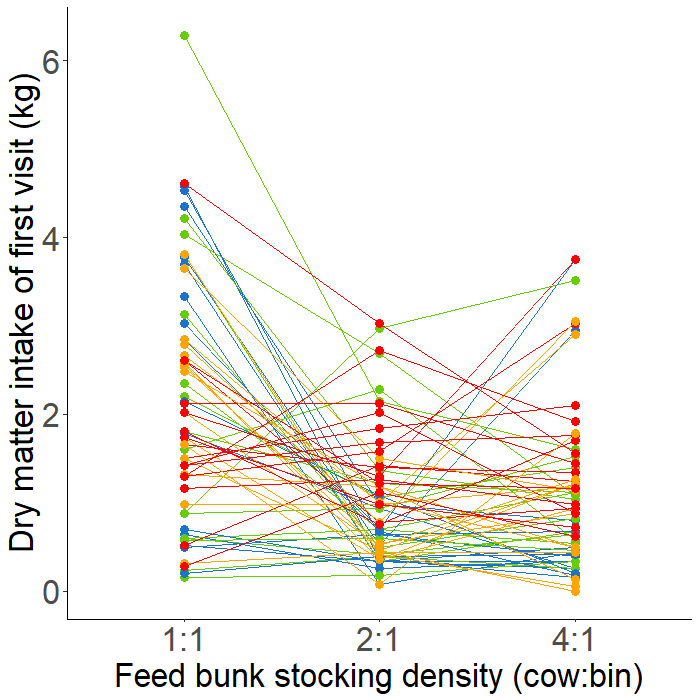

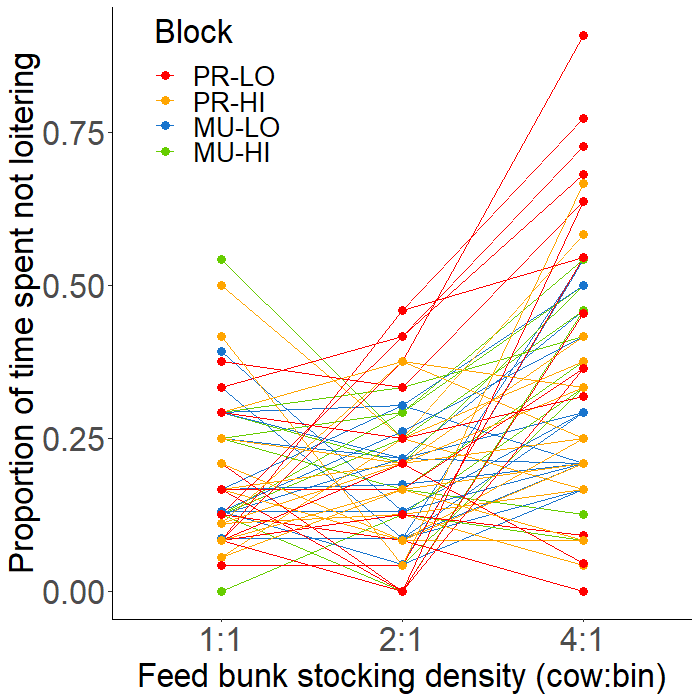


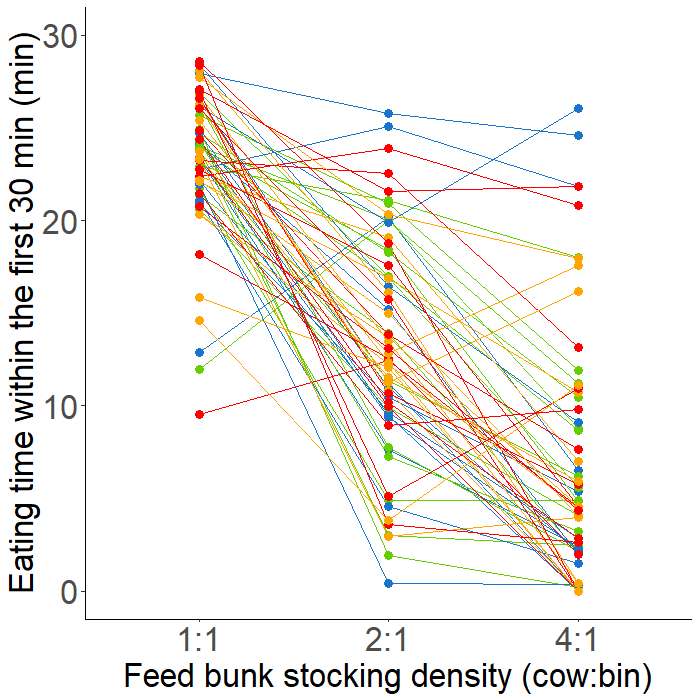

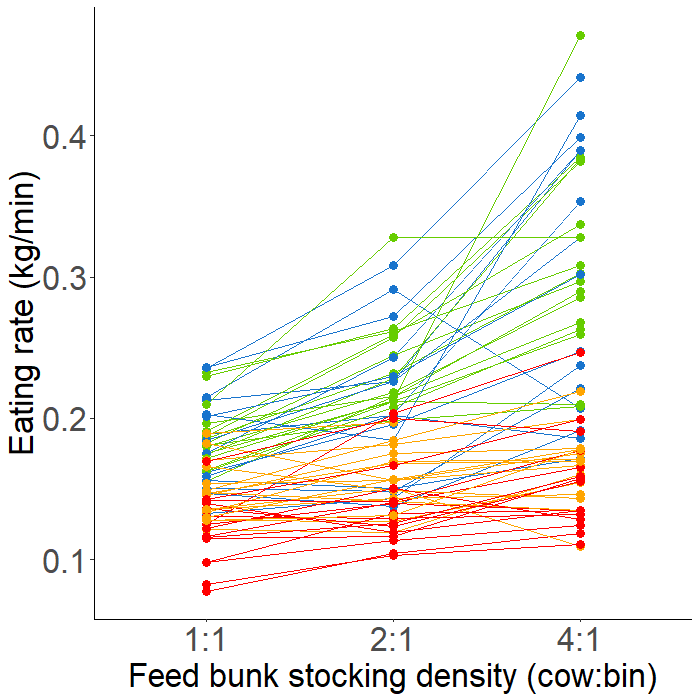

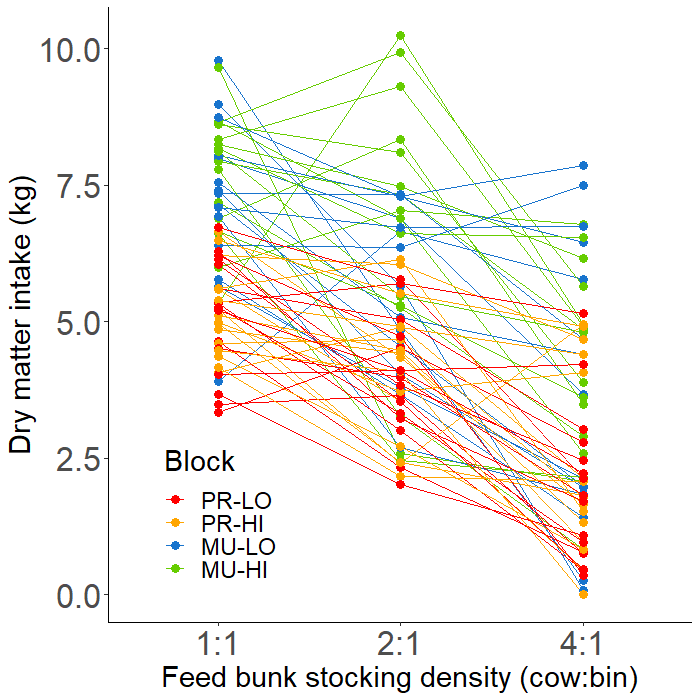

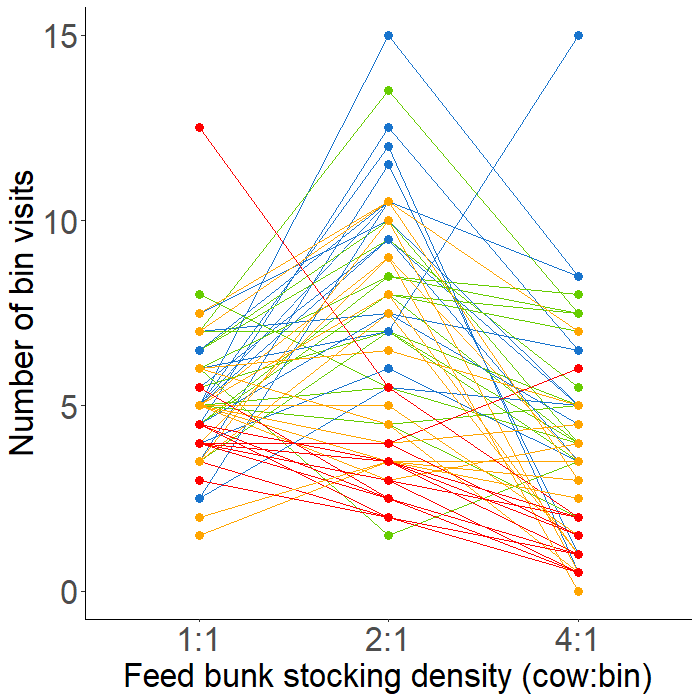


**(I)**

**(J)**

**(K)**

**(L)**


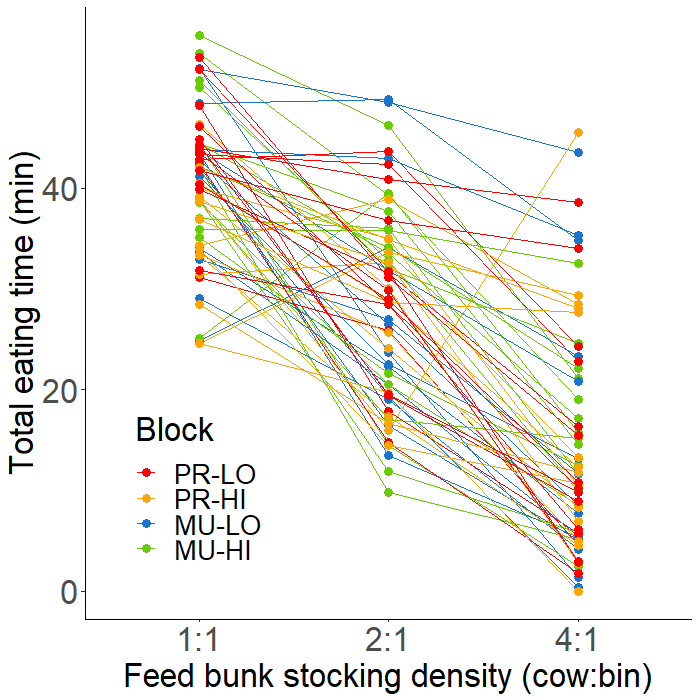


**(M)**
